# Gregariousness does not vary with geography, developmental stage, or group relatedness in feeding redheaded pine sawfly larvae

**DOI:** 10.1002/ece3.2952

**Published:** 2017-04-17

**Authors:** John W. Terbot, Ryan L. Gaynor, Catherine R. Linnen

**Affiliations:** ^1^ Department of BiologyUniversity of KentuckyLexingtonKYUSA

**Keywords:** behavioral assay, behavioral development, Diprionidae, feeding aggregations, gregariousness

## Abstract

Aggregations are widespread across the animal kingdom, yet the underlying proximate and ultimate causes are still largely unknown. An ideal system to investigate this simple, social behavior is the pine sawfly genus *Neodiprion*, which is experimentally tractable and exhibits interspecific variation in larval gregariousness. To assess intraspecific variation in this trait, we characterized aggregative tendency within a single widespread species, the redheaded pine sawfly (*N. lecontei*). To do so, we developed a quantitative assay in which we measured interindividual distances over a 90‐min video. This assay revealed minimal behavioral differences: (1) between early‐feeding and late‐feeding larval instars, (2) among larvae derived from different latitudes, and (3) between groups composed of kin and those composed of nonkin. Together, these results suggest that, during the larval feeding period, the benefits individuals derive from aggregating outweigh the costs and that this cost‐to‐benefit ratio does not vary dramatically across space (geography) or ontogeny (developmental stage). In contrast to the feeding larvae, our assay revealed a striking reduction in gregariousness following the final larval molt in *N. lecontei*. We also found some intriguing interspecific variation: While *N. lecontei* and *N. maurus* feeding larvae exhibit significant aggregative tendencies, feeding *N. compar* larvae do not aggregate at all. These results set the stage for future work investigating the proximate and ultimate mechanisms underlying developmental and interspecific variation in larval gregariousness across *Neodiprion*.

## 1 Introduction

Aggregations, or spatial groupings of organisms, are widespread in nature and occur across diverse taxa (Krause & Ruxton, 2002; Parrish & Edelstein‐Keshet, 1999; Prokopy & Roitberg, 2001). While some aggregations are passive, arising as a consequence of features of the landscape that lead to clumped distributions (e.g., Carpenter, 1954; Schartel & Schauber, 2016), many aggregations stem from individuals actively seeking out and maintaining contact with conspecifics (e.g., Costa & Louque, 2001; Jeanson et al., 2005; Schmuck, 1987). To understand these aggregative behaviors, we must investigate both their proximate (developmental, physiological, and molecular mechanisms) and ultimate (adaptive function and evolutionary history) causes (Tinbergen, 1963). Integration of these distinct perspectives is most easily accomplished with (1) experimentally tractable organisms (i.e., can be reared, crossed, and manipulated in the laboratory) with interesting behavioral variation, and (2) simple and reliable assays for quantifying those behaviors (e.g., Ame, Rivault, & Deneubourg, 2004; Broly, Mullier, Deneubourg, & Devigne, 2012; De Bono & Bargmann, 1998; Fujiwara, Sengupta, & McIntire, 2002; Jeanson et al., 2003, 2005; Osborne et al., 1997; Sokolowski, Pereira, & Hughes, 1997; Wu et al., 2003). In this study, we introduce a potentially powerful system for investigating both the proximate and ultimate causes of behavioral variation and describe an assay for quantifying one variable behavior involved in aggregation, larval aggregative tendency.

*Neodiprion* (Hymenoptera: Diprionidae) is a Holarctic genus of ~50 sawfly species, all of which specialize on host plants in the family Pinaceae (Linnen & Smith, 2012; Wallace & Cunningham, 1995). Because many species in the genus are forestry pests, *Neodiprion* life histories have been studied in great detail. These studies have revealed a remarkable amount of inter‐ and intraspecific variation in a wide range of traits, including host preference, oviposition pattern, larval color, overwintering stage, and larval gregariousness (Atwood, 1962; Baker, 1972; Coppel & Benjamin, 1965; Knerer, 1984, 1993; Larsson, Björkman, & Kidd, 1993). In addition to harboring variation in many interesting traits, *Neodiprion* are experimentally tractable. They can be manipulated in the laboratory and in the field, and many different interspecific crosses are possible (Kraemer & Coppel, 1983; Linnen & Farrell, 2007; Ross, 1961; personal observation). Moreover, a molecular phylogeny is available for the genus (Linnen & Farrell, 2008a,b), and there are a growing number of genomic resources, including an assembled and annotated genome for the redheaded pine sawfly (*N. lecontei*; Vertacnik, Geib, & Linnen, 2016), a linkage map and genome assemblies for all 20 species in the eastern North American “*Lecontei*” clade (unpublished data). Together, the well‐described natural history, extensive variation, and growing set of genetic and genomic tools will facilitate investigations into the proximate and ultimate causes of many different types of traits.

**Figure 1:**
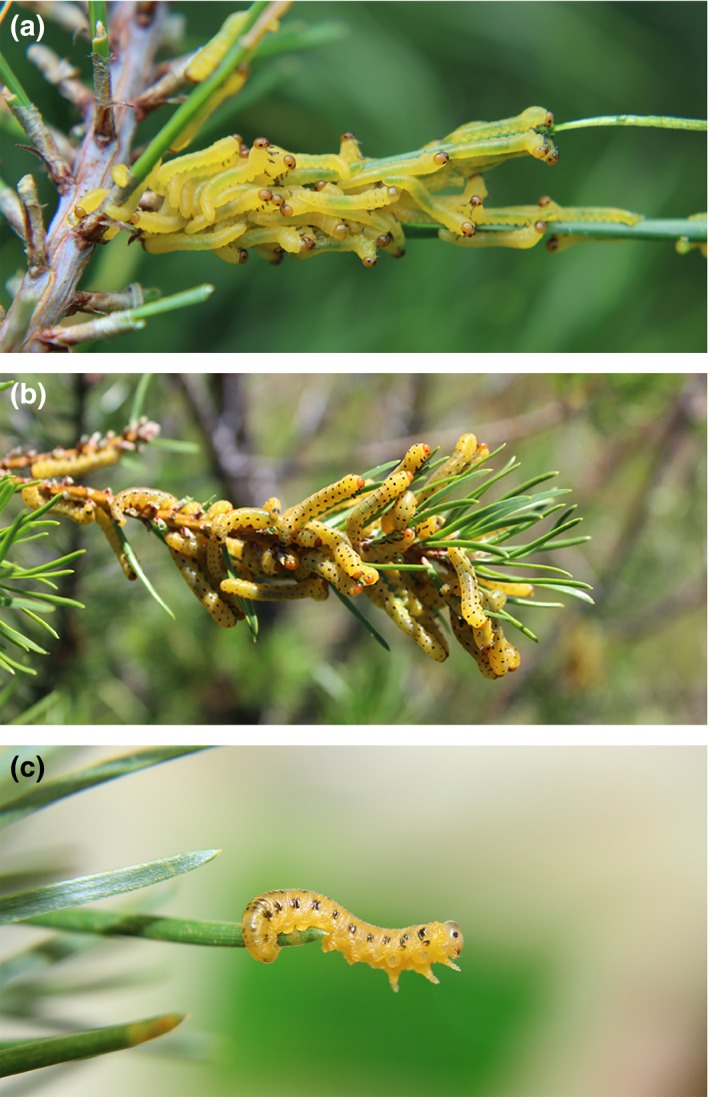
Developmental variation in larval morphology in *Neodiprion lecontei*. Representative photographs of early‐feeding (a), late‐feeding (b), and nonfeeding (c) instars. Photographs by R. Bagley

Importantly, *Neodiprion* larvae exhibit intriguing developmental and interspecific variation in their tendency to aggregate. While larvae of many *Neodiprion* species have been categorized as “gregarious” and form conspicuous feeding aggregations in the field, larvae of several species that do not form large aggregations are categorized as “solitary” or “intermediate” (Larsson et al., 1993). Moreover, the tendency to aggregate appears to change over the course of development. For example, all *Neodiprion* species have a morphologically and behaviorally distinct final, nonfeeding instar (Figure 1; Ghent, 1960; Hetrick, 1959; Smith, 1993). During this stage, any aggregative tendency disappears as the larva wanders from the group to find an appropriate site to spin a cocoon in which to pupate. Additionally, in at least some *Neodiprion* species (e.g., *N. tsugae*,* N. abietis,* and *N. abbotii*), larval aggregative tendencies appear to decline in late‐feeding instars (Anstey, Quiring, & Ostaff, 2002; Furniss & Dowden, 1941; Hetrick, 1956; Hopping & Leech, 1936; Rose & Lindquist, 1994).

To understand why some *Neodiprion* species and life stages tend to aggregate and others do not, we must consider the costs and benefits of aggregating. Costs of gregariousness in pine sawfly larvae (and other externally feeding folivores) include increased disease risk (Bird, 1955; Fletcher, 2009; Hochberg, 1991; Mohamed, Coppel, & Podgwaite, 1985; Young & Yearian, 1987), increased predation risk (Bertram, 1978; Lindstedt, Huttunen, Kakko, & Mappes, 2011; Vulinec, 1990), and competition for resources (Pimentel, Santos, Ferreira, & Nilsson, 2012; Prokopy, Roitberg, & Averill, 1984). Proposed benefits of gregariousness in pine sawfly larvae include thermoregulation (Codella & Raffa, 1993; Fletcher, 2009; Joos, Casey, Fitzgerald, & Buttemer,^1988^; Klok & Chown, 1999; McClure, Cannell, & Despland, 2011; Seymour, 1974), enhancement of group defense (Bertram, 1978; Codella & Raffa, 1993; McClure & Despland, 2011; McClure, Ralph, & Despland, 2011; Pulliam & Caraco, 1984; Tostowaryk, 1972), and increased foraging efficiency/improved ability to overcome plant defenses (Codella & Raffa, 1993; Despland & Le Huu, 2007; McClure, Morcos, & Despland, 2013; Stamp & Bowers, 1990; Tsubaki & Shiotsu, 1982; Young & Moffett, 1979). If there is heritable variation in gregariousness and the costs and benefits of aggregating vary among populations and species, natural selection is expected to produce intra‐ and interspecific variation in aggregation behavior. Additionally, whenever aggregation costs outweigh its benefits, natural selection should favor a complete loss of gregariousness. Testing these predictions requires objective methods for quantifying aggregative behaviors and for distinguishing between gregarious and nongregarious behavior.

To date, most descriptions of larval gregariousness in *Neodiprion* have been qualitative, assigning species to different behavioral categories (i.e., “gregarious,” “intermediate,” and “solitary”) on the basis of the size of typical larval aggregations encountered in the field (Larsson et al., 1993). One problem with this approach is that colony size depends not only on the behavior of aggregating larvae, but also on the behavior of ovipositing females. For example, while females of some species tend to lay all of their eggs on a single branch terminus, others distribute their eggs across multiple hosts (Atwood, 1962; Baker, 1972; Coppel & Benjamin, 1965; Knerer, 1984, 1993; Larsson et al., 1993). Because female oviposition behavior may be shaped by selection pressures that are distinct from those shaping larval behavior (Nufio & Papaj, 2012; Scheirs, De Bruyn, & Verhagen, 2000; Scheirs, Jordaens, & De Bruyn, 2005), it is important that we disentangle the contributions of adult and larval behaviors to larval aggregation size. Additionally, because qualitative categories may miss ecologically relevant behavioral variation, it is essential that we quantify these behaviors. To these ends, we describe here a simple quantitative assay of larval aggregative tendency under artificial, but highly repeatable, conditions.

To evaluate our assay, we focused on the redheaded pine sawfly, *N. lecontei*. Although our ultimate goal is to assay larval behavior across the genus *Neodiprion*, we chose to focus on this species first because its life history and highly gregarious behavior are especially well described in the literature (Benjamin, 1955; Codella & Raffa, 1993, 1995a,b; Costa & Louque, 2001; Flowers & Costa, 2003; Wilson, Wilkinson, & Averill, 1992). *Neodiprion lecontei* is widely distributed in eastern North America, where it occurs on multiple pine species (Linnen & Farrell, 2010; Wilson et al., 1992). After mating, adult females use their saw‐like ovipositors to embed their eggs into the host plant needles. Usually, an individual female will lay her entire complement of ~100–150 eggs in adjacent needles in a single branch terminus (Benjamin, 1955; Wilson et al., 1992). Upon hatching, larvae form aggregations and feed in groups until they molt into the final, nonfeeding instar (Figure 1). When a branch is defoliated, larvae migrate in small groups to a new feeding site, where they recoalesce. Colony migration appears to be mediated both by chemical cues deposited by the migrating larvae (which serve to orient larvae to the new feeding site) and by tactile cues from the larvae themselves (which reinforce feeding site selection; Costa & Louque, 2001; Flowers & Costa, 2003). Additionally, isolated *N. lecontei* larvae become highly agitated and exhibit increased wandering behavior, presumably in search of a feeding aggregation to join (Kalin & Knerer, 1977). Thus, while initial colony size may be attributable to the behavior of ovipositing females (Codella & Raffa, 1995a), detection of and response to larval cues maintain colony cohesion over the course of development (Costa & Louque, 2001; Flowers & Costa, 2003).

Together, these previously published accounts of *N. lecontei* behavior provide us with testable predictions that we can evaluate with a quantitative assay. First, we asked how larval aggregative tendency changes over the course of the larval feeding period. In diprionid sawflies, early‐instar larvae may experience difficulty establishing feeding incisions on tough pine foliage; in aggregations, so long as some individuals are able to make a feeding incision, the group can benefit (Ghent, 1960; but see Kalin & Knerer, 1977). However, older larvae have no difficulty feeding; thus, if there are not additional benefits to group‐living, its costs may favor colony splitting (Codella & Raffa, 1993; Coppel & Benjamin, 1965). Based on existing natural history literature and our own experience, we predicted that all feeding instars would aggregate. However, if there is a large reduction in the net benefit of aggregating over the course of larval development, we expected to see a corresponding decrease in larval aggregative tendency. Second, we asked how larval aggregative tendency changed between feeding and nonfeeding instars. Because nonfeeding instars disperse to spin cocoons, we expected a complete loss of aggregative tendency in the final, nonfeeding instars.

Third, we asked how relatedness among group members impacts larval aggregative tendency. If aggregating is costly to individual sawfly larvae (e.g, Bird, 1955; Lindstedt et al., 2011; Mohamed et al., 1985; Young & Yearian, 1987), kin selection theory predicts that kin groups will have elevated aggregative tendency compared to nonkin groups. Alternatively, we would expect aggregative tendency to be unaffected by the relatedness of group members if: the direct benefits of aggregating outweigh its costs; individual larvae are unable to distinguish between kin and nonkin; or the costs of kin‐based discrimination are too high.

Finally, after exploring how *N. lecontei* behavior changes with development and group composition, we apply our assay to multiple *N. lecontei* populations and two additional *Neodiprion* species (one “gregarious” species and one “solitary” species) to gain a first glimpse into levels of interpopulation and interspecific variation in larval behavior in the genus. Together, our results lay the groundwork for future studies, while also providing insights into both the proximate and ultimate mechanisms underlying larval aggregative tendency.

## 2 Materials and Methods

### 2.1 Collection and rearing information

Sawfly larvae used in our experiments were either wild‐caught or derived from colonies that we reared for no more than two generations in the laboratory using our standard laboratory protocols (described in more detail in Bagley et al., 2017; Harper, Bagley, Thompson, & Linnen, 2016). Briefly, we transported wild‐caught larval colonies to the laboratory in brown paper bags. Upon arrival, we transferred each larval colony to a plastic box with a mesh top (32.4 × 17.8 × 15.2 cm) and fed them clipped pine foliage from their natal host species as needed until they had spun cocoons. We stored cocoons individually in size “0” gelatin capsules and checked daily for emergence. We stored emerged adults at 4°C until needed. To produce the next generation of larvae, we released adult females (either mated or unmated, see below) into large mesh cages (35.6 × 35.6 × 61 cm) containing *Pinus banksiana* seedlings. Once eggs had hatched and larvae had consumed the seedling foliage, we transferred them to plastic boxes and reared them on clipped *P. banksiana* foliage as described above.

Like all hymenopterans, *Neodiprion* have haplodiploid sex determination; mated females produce diploid daughters and haploid sons and unmated females produce haploid males only (Harper et al., 2016; Heimpel & de Boer, 2008). For our experiments, we used larvae derived from both unmated and mated females. Whenever possible, we used the haploid male offspring of unmated females to minimize possible noise stemming from sex‐based differences in behavior. However, for some experiments, families from unmated females were not available. Because we cannot easily differentiate between female and male larvae, families derived from mated females likely contained a mixture of both sexes. We provide more detailed information on the source and rearing history of larvae for each experiment below and in Table 1.

**Table 1:** Collection data for *Neodiprion* colonies

Colony ID^a^	Species	Date of collection	Nearest City, State	Host plant	Latitude, Longitude
RB261	*Neodiprion lecontei*	7/17/2013	Grayling, MI	*Pinus banksiana*	44.65689, −84.6958
LL031	*N. lecontei*	8/14/2013	Piscataway, NJ	*P. sylvestris*	40.54955, −74.4308
RB244	*N. lecontei*	7/16/2013	Bitely, MI	*P. banksiana*	43.79322, −85.74
RB316	*N. lecontei*	8/7/2013	Orange Springs, FL	*P. palustris*	29.50772, −81.8598
RB335	*N. lecontei*	8/22/2013	Lexington, KY	*P. elliottii*	38.014, −84.504
RB380, RB381, RB383, RB384	*N. lecontei*	7/15/2015	Bitely, MI	*P. banksiana*	43.7675, −85.7403
RB397, RB398, RB399, RB400	*N. lecontei*	7/17/2015	Necedah, WI	*P. banksiana*	44.15611, −90.1322
NS037	*N. maurus*	6/17/2014	Rhinelander, WI	*P. banksiana*	45.66427, −89.4919
NS043	*N. lecontei*	7/2/2014	Spooner, WI	*P. banksiana*	45.82233, −91.8884
CN001 (NS174)	*N. compar*	8/15/2015	Hawk Junction, ON	*P. banksiana*	48.04558, −84.5494
CN001 (NS182)	*N. compar*	8/17/2015	Gurney, WI	*P. banksiana*	46.50895, −90.5027
CN002 (NS175)	*N. compar*	8/15/2015	Hawk Junction, ON	*P. banksiana*	48.02968, −84.6513
CN002 (NS184)	*N. compar*	8/17/2015	Glidden, WI	*P. banksiana*	46.11489, −90.5511
CN003 (NS176)	*N. compar*	8/15/2015	White River, ON	*P. banksiana*	48.54371, −85.1911
CN003 (NS178)	*N. compar*	8/16/2015	Mokomon, ON	*P. banksiana*	48.41605, −89.6412
CN003 (NS168)	*N. compar*	8/13/2015	Petawawa, ON	*P. banksiana*	45.92631, −77.3254
CN004 (NS169)	*N. compar*	8/13/2015	Petawawa, ON	*P. banksiana*	45.93154, −77.3333
CN004 (NS170)	*N. compar*	8/14/2015	Onaping, ON	*P. banksiana*	46.62311, −81.4552
CN004 (NS172)	*N. compar*	8/14/2015	Gogama, ON	*P. banksiana*	47.46476, −81.8467
CN004 (NS174)	*N. compar*	8/15/2015	Hawk Junction, ON	*P. banksiana*	48.04558, −84.5494

a Each colony ID corresponds to a unique larval colony (or individual) collected in the field. When multiple colonies were collected at the same location at the same time, multiple colony IDs are given. Because *N. compar* videos combined larvae from different locations, two IDs are given. The first ID refers to how larvae were grouped into one of 4 videos (CN001‐CN004); the second, in parentheses, refers to the original collection ID (NS168, NS169, NS170, NS172, NS174, NS175, NS176, NS178, NS192, or NS182).

### 2.2 Video assays of larval aggregative tendency

**Figure 2:**
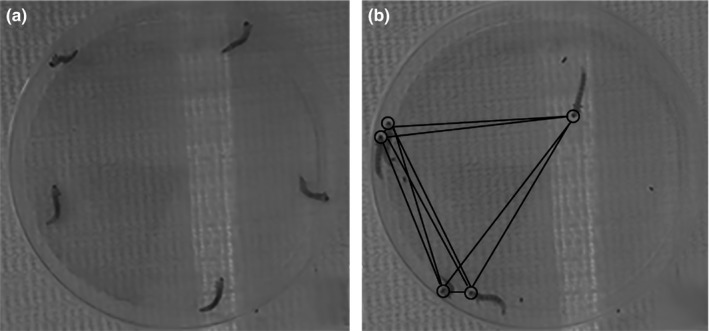
Gregarious assay test arena. (a) Equidistant placement of larvae at the start of the video. (b) Image taken from the middle of a larval video. Circles indicate the location of head capsules, and lines indicate pairwise distances

To measure larval aggregative tendency, we developed a video assay. Prior to the start of each video, we spaced larvae equidistantly along the perimeter of a 14.5 cm petri dish (Figure 2). The number of larvae per video varied from 2 to 8, depending on the experiment, and no larvae were used in more than one video. For our first set of assays, we used mixed‐sex larvae derived from mated mothers from Grayling, MI (from RB261, Table 1). We recorded each group of larvae for 90 min on either a Logitech or Microsoft webcam connected to a Lenovo Ideapad laptop. We recorded all videos in an environmental room at 22°C and 70% relative humidity. For each video, we then used the program Video Image Master Pro (A4Video 2016) to extract one frame every fifteen seconds, for a total of 360 frames per video. Based on preliminary analyses of different intervals ranging from 15 to 1,800 s, we further reduced the sampling for each video to one frame every 180 s (30 frames per video). We chose this sampling frequency because it reduced data processing time, while yielding results indistinguishable from shorter intervals.

For each video frame, we manually selected the position of each larval head capsule and calculated all pairwise distances using a custom Java application. Although video scoring was not blind with respect to our treatments, the objective nature of our data collection (clicking on the physical position of head capsules) provides minimal opportunity for observer bias to influence our results. After videos were scored, we used a combination of Microsoft Excel 2013 (Microsoft 2012) and a custom Perl script to calculate the mean pairwise distances for the entire video as well as subsets of the video. Analysis of the full video yielded 30 pairwise distances per video.

Differences in larval mobility could influence pairwise larval distances and, thus, affect our measurement of larval aggregative tendency. We therefore used two approaches to minimize the impact of larval mobility. First, we visually examined each video to ensure all larvae were in good condition and moving freely during the recording. Second, because frames at the start of the video reflected experimental spacing rather than larval behavior and because handled larvae sometimes become agitated (Costa & Louque, 2001), we examined a large number of videos to see how pairwise distances changed over time and to determine how long it takes for pairwise distances to stabilize. Based on these preliminary analyses, we discarded the first 12 frames from every video as an acclimation period. We then averaged the remaining 18 frames to produce a single summary statistic for each video, which we refer to as the mean pairwise distance. Overall, videos with smaller mean pairwise distances indicate that larvae tended to remain closer to each other and thus can be described as having a higher aggregative tendency. We log‐transformed (natural log) pairwise distances prior to statistical analysis to reduce the impact of outliers and to satisfy the assumptions of the statistical tests used.

The host‐free petri dish environment used in our assays is obviously very different from conditions under which larvae aggregate in nature. Nevertheless, our assay will measure—under these simple conditions—what we refer to as “larval aggregative tendency.” Specifically, the aggregative tendency of a particular group of larvae (quantified as the average mean pairwise distance of the larvae following the acclimation period) will reflect a combination of: (1) the tendency of the larvae to form an aggregation in the first place, and (2) the cohesiveness of the aggregation once formed. The primary benefits of this assay are that it is fast, simple, and can be applied in a consistent manner to any group of larvae, facilitating comparisons among different populations and species. In the discussion, we consider possible limitations and extensions of our assay.

### 2.3 Generating a model of random dispersal

To generate the expected distribution of pairwise distances under the null hypothesis that larvae distribute themselves randomly in the petri dish (i.e., do not actively aggregate or disperse), we used a custom Java application to perform a series of simulations that mimicked our sampling procedure. For each of the experimental group sizes that we used (2, 5, 6, and 8 larvae), we simulated 100 videos by randomly placing the corresponding number of points (2, 5, 6, or 8) in a virtual 14.5‐cm circular arena. To mimic our subsampling, we repeated this process 30 times (“frames”) per “video” and calculated mean pairwise distance across all frames. We then calculated a 95% confidence interval from the 100 simulated mean pairwise distances.

### 2.4 Effect of developmental stage on aggregative tendency

To determine the impact of developmental stage on the aggregative tendency of *N. lecontei* larvae and to assess optimal group size for our assays, we recorded videos for all possible combinations of three developmental stages (early‐feeding instars, late‐feeding instars, and nonfeeding instars) and three group sizes (2, 5, and 8 larvae). *Neodiprion lecontei* males have five feeding instars, while *N. lecontei* females have six; both sexes have a single nonfeeding instar. We determined developmental stage based on reliable changes in size and color that accompany larval development (Wilson et al., 1992). In our analysis, we considered second and third instars to be “early‐feeding instars.” These larvae had head capsules ≤1.05 mm (0.44–1.03 mm) and had not yet developed mature coloration, which consists of a reddish orange head capsule with a black ring around each eye and up to four paired rows of black spots (Wilson et al., 1992; Figure 1a). We considered fourth through sixth instars to be “late‐feeding instars.” These larvae had fully developed color patterns and head capsules ≥1.5 mm (1.5–1.86 mm; Figure 1b). “Nonfeeding instars” were easily identifiable due to their distinct coloration pattern (pale cream to yellow body color and head capsule, distinct spotting pattern; Ghent, 1960; Hetrick, 1959; Smith, 1993; Figure 1c). For these experiments, we used mixed‐sex larvae produced by mated females from the Grayling, MI laboratory colony (from RB261; Table 1).

For each combination of group size and developmental stage, we recorded five videos, for a total of 45 videos. We processed these videos as described above (one frame every 180 s, with the first 2,160 s—or 12 frames—discarded as an acclimation period). We then averaged all pairwise distances from each video and log‐transformed (natural log) this value to obtain the video's log mean pairwise distance. We analyzed these values with an ANOVA, followed by post hoc *t* tests to determine which life stages differed significantly. Finally, we compared each life stage and group‐size combination to the randomly generated distribution described above. Because distances recorded from different larval group sizes are not directly comparable (i.e., the maximum possible pairwise distance declines as group size increases), we analyzed each group size separately. We performed all ANOVA and Tukey HSD tests in JMP10 (SAS Institute 2012).

### 2.5 Effect of relatedness on aggregative tendency

To determine whether the relatedness of larvae impacts their tendency to aggregate, we videotaped larval behavior under three treatments: (1) all larvae derived from the same mother (brothers), (2) an equal mix of larvae from two mothers from the same population (nonsiblings, but possibly related), (3) an equal mix of larvae from two mothers from different populations (nonrelatives). For these assays, we used haploid male larvae produced by virgin mothers derived from two populations: one near Bitely, Michigan (from RB380, RB381, RB383, RB384; Table 1), and another near Necedah, Wisconsin (from RB397, RB398, RB399, RB400; Table 1). For each treatment, we recorded 14–17 videos of six late‐feeding instar larvae, for a total of 46 videos (*N* = 17, 14, and 15 videos for same mother, different mother/same population, different mother/different population, respectively). We used an ANOVA to determine if relatedness had an effect on log‐transformed mean pairwise distance (natural log). Again, we compared each treatment to our random, null distribution using Tukey HSD.

### 2.6 Intraspecific variation in aggregative tendency

To assess the extent to which different *N. lecontei* populations vary in their aggregative tendency, we recorded videos of larvae from five different locations (Table 1): Bitely, MI (from RB244; *N *= 29 videos); Grayling, MI (from RB261; *N *= 32 videos); Orange Springs, FL (from RB316; *N* = 19 videos); Lexington, KY (from RB335; *N* = 18 videos); and Piscataway, NJ (from LL031; *N* = 21 videos). These populations were chosen because they provide a representative sample of the geographical range and genetic diversity of *N. lecontei*. In particular, all three major genetic clusters identified via a population genomic analysis are represented in our sample (Bagley et al., in press). To obtain larvae for video analyses, we reared the haploid male offspring from 15 to 18 virgin females per population. For these assays, we used eight late‐feeding instars per video. We log‐transformed (natural log) pairwise distances and used ANOVAs to determine whether aggregative tendency differed among: populations, genetic clusters, or by latitude. Populations, both separately and combined by genetic cluster, were also compared to the random, null model using Tukey HSD.

### 2.7 Interspecific variation in aggregative tendency

To determine how aggregative tendency of *N. lecontei* larvae compares with other *Neodiprion* species, we recorded videos of two other *Neodiprion* species. One of these species (*N. maurus*) has been categorized as “gregarious,” while the other species (*N. compar*) has been categorized as “solitary” (Larsson et al., 1993; Wilson, 1977). For these assays, we used five late‐feeding instar larvae per video, all of which were wild‐caught. Our sample sizes for each species were as follows: *N* = 7 for *N. maurus* (from NS037; Table 1); *N* = 8 for *N. lecontei* (from NS043; Table 1); *N* = 4 for *N. compar* (from multiple colonies, Table 1). We note that because *N. compar* is rarely found in groups in nature, we had to combine individuals from multiple sites and our sample sizes were limited compared to other species. We used ANOVAs to compare log‐transformed (natural log) mean pairwise differences among species, followed by post hoc, pairwise Tukey HSD tests. To further evaluate previous designations of “gregarious” and “solitary,” we compared data from each of these species to our simulated random distribution using a Tukey HSD test.

## 3 Results

### 3.1 Effect of developmental stage and number of larvae on aggregative tendency

**Figure 3:**
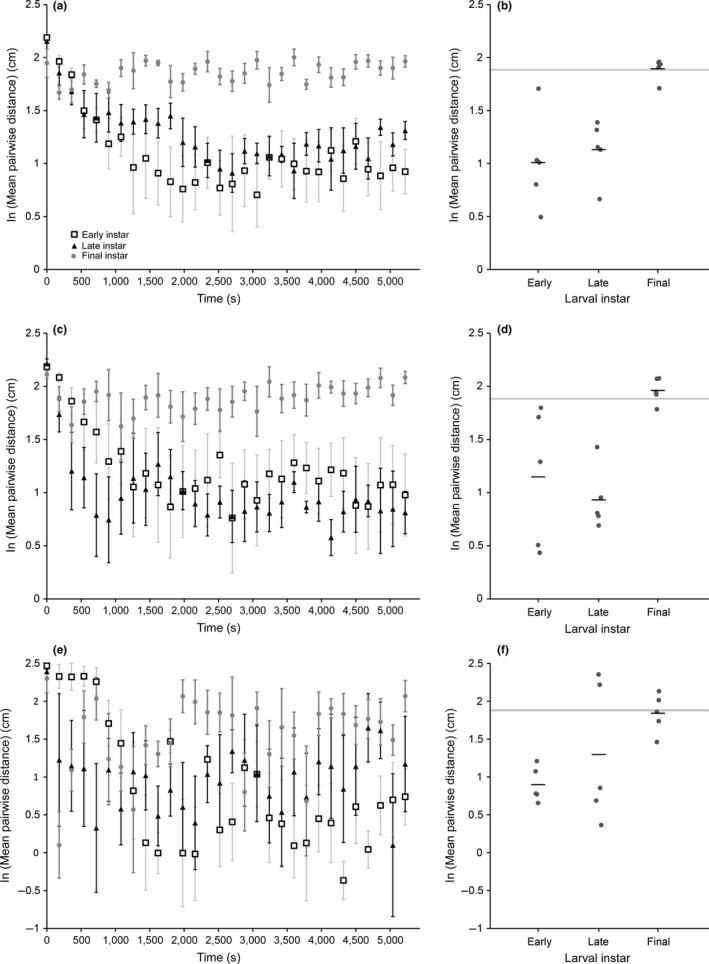
Impact of group size and developmental stage on aggregative tendency. Log‐transformed pairwise distances (natural log) estimated from videos using eight (a, b), five (c, d), or two (e, f) larvae. In (a), (c), and (e), points are the mean (±SEM) average pairwise distances computed from a single time point (video frame) for a particular developmental stage: early‐feeding instars (open squares), late‐feeding instars (black triangles), nonfeeding instars (light gray circles). Note that pairwise distances tend to stabilize by ~2,000 s. In (b), (d), (e), each point represents the log‐transformed mean pairwise distance (natural log) calculated from a single video, following a 2,160‐s acclimation period. Horizontal bars represent the overall average for each life stage. The light gray bar in (b), (d), and (e) represents the 95% confidence interval for mean pairwise distance estimated via simulation under a model of random larval distribution for the respective number of larvae. Whereas early‐ and late‐feeding instars aggregate significantly more than the null model, final instars do not aggregate

In total, we recorded 45 videos of various combinations of larval group size and developmental stage. Before analyzing these data, we first confirmed that our 2,160‐s acclimation period was sufficient for larval behavior to stabilize. As illustrated in Figure 3a,c,e, pairwise distances tend to stabilize by approximately 1,200–1,800 s for all treatments.

When we condensed each video down to a single log‐transformed (natural log) mean pairwise distance (for postacclimation period only), we found that developmental stage had a pronounced impact on aggregative tendency, but that its effects were partially dependent on the number of larvae in the assay (Figure 3b,d,f). We found that developmental stage significantly impacted aggregative tendency in the 5‐ and 8‐larvae videos (5‐larvae videos: ANOVA, *F*_2,12_ = 8.4885, *p* = .0050; 8‐larvae videos: ANOVA, *F*_2,12_ = 11.9256, *p* = .0014). In both cases, this difference was attributable to a decrease in aggregative tendency (i.e., an increase in average pairwise distance) that occurred in the final, nonfeeding instar (5‐larvae videos Tukey HSD: early‐feeding vs. late‐feeding, *p* = .6979; early‐feeding vs. nonfeeding, *p* = .0237; late‐feeding vs. nonfeeding, *p* = .0055; 8‐larvae videos Tukey HSD: early‐feeding vs. late‐feeding, *p* = .8119; early‐feeding vs. nonfeeding, *p* = .0019; late‐feeding vs. nonfeeding, *p* = .0057). By contrast, the impact of developmental stage on aggregative tendency was not significant in the 2‐larvae videos (ANOVA, *F*_2,12_ = 3.4549, *p* = .0653). We note, however, that the overall patterns are the same (nonfeeding instar is less gregarious than feeding instars) in the 2‐larvae videos. Our observed lack of significance for the 2‐larvae treatment likely stems from a greater intervideo variation (Figure 3e), suggesting that larger group sizes (e.g., five or more larvae) may yield more reliable results than smaller group sizes.

We also compared our observed pairwise distances to those expected under the null hypothesis that larvae distribute themselves randomly throughout the arena. For all group sizes, the early‐feeding and late‐feeding instars had significantly smaller pairwise differences than the random model (2‐larvae videos Tukey HSD: early‐feeding vs. random *p* < .0001; late‐feeding vs. random *p* < .0001; 5‐larvae videos Tukey HSD: early‐feeding vs. random *p* < .0001; late‐feeding vs. random *p* < .0001; 8‐larvae videos Tukey HSD: early‐feeding vs. random *p* < .0001; late‐feeding vs. random *p* < .0001). Together, these results confirm that *N. lecontei* feeding instars are gregarious. Additionally, for the 2‐ and 8‐larvae videos, nonfeeding instars did not differ significantly from the random model (2‐larvae Tukey HSD: *p* = .3498; 8‐larvae Tukey HSD: *p* = .4972). By contrast, nonfeeding instars from the 5‐larvae videos appeared to have greater pairwise distances than expected under the random model (Tukey HSD: *p* = .0001). Together, these results suggest that while *N. lecontei* feeding instars have a strong behavioral tendency to aggregate, nonfeeding instars either ignore or actively avoid one another.

### 3.2 Effect of relatedness on aggregative tendency

**Figure 4:**
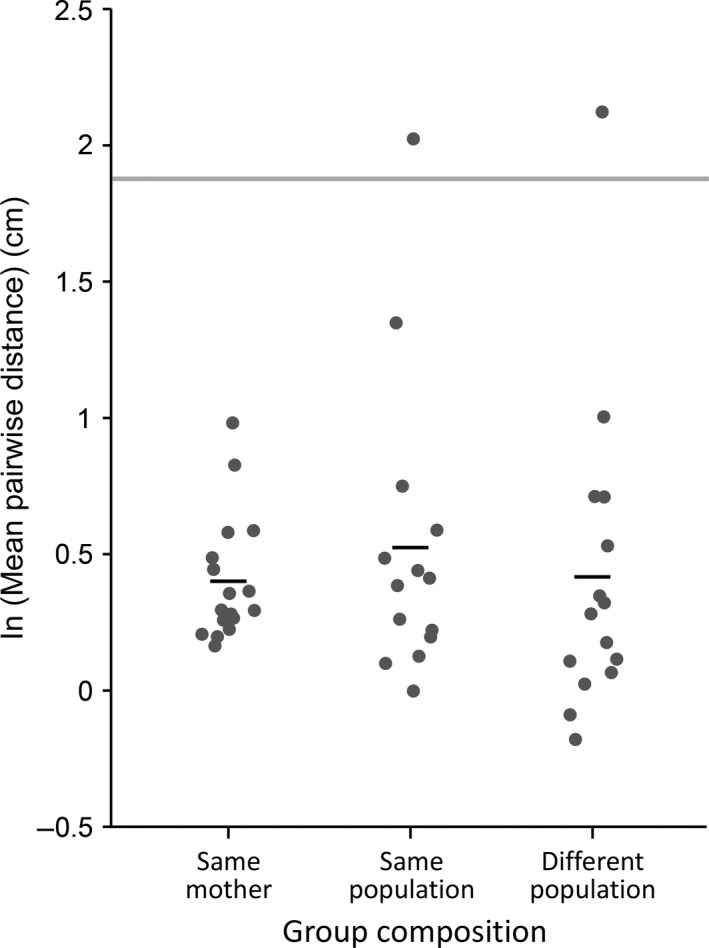
Impact of relatedness on aggregative tendency. Each dark gray circle represents the log‐transformed (natural log) mean pairwise distances estimated from a single video of six late‐feeding instar larvae (following a 2,160‐s acclimation period), and black bars represent the overall mean for each treatment. The light gray bar represents the 95% confidence interval for the mean pairwise distance between six larvae estimated via simulation under a model of random larval distribution. Relatedness had no impact on larval aggregative tendency

In our experiments, relatedness had no detectable impact on the aggregative tendency of larvae (Figure 4, ANOVA, *F*_2,43_ = 0.3045; *p* = .7391). Moreover, all three treatments were significantly more aggregative than the random model (Tukey HSD: *p *< .0001 for all comparisons).

### 3.3 Intraspecific variation in aggregative tendency

**Figure 5:**
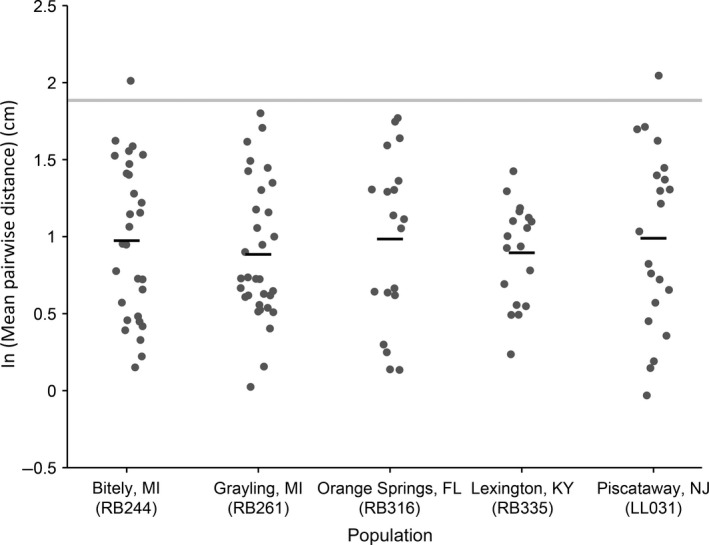
Intraspecific variation in aggregative tendency. Each dark gray circle represents the log‐transformed (natural log) mean pairwise distances estimated from a single video of eight late‐feeding instar, male larvae (following a 2,160‐s acclimation period); black bars represent the overall mean for each population. The light gray bar represents the 95% confidence interval for the mean pairwise distance between eight larvae estimated via simulation under a model of random larval distribution. Compared to within‐population variation, between‐population variation was minimal

Examination of aggregative tendency of late‐feeding instars sampled from diverse *N. lecontei* populations revealed substantial within‐population variation, but very little variation among populations (Figure 5). Population of origin did not significantly affect aggregative tendency (ANOVA, *F*_4,114_ = 0.2662, *p* = .8991). We also did not detect any differences when we lumped populations according to their membership in one of three genetic clusters (ANOVA, *F*_2,116_ = 0.1014; *p* = .9037), nor did we detect any relationship between population latitude and aggregative tendency (ANOVA, *F*_1,117_ = 0.1644; *p* = .6859). Finally, each of the five populations and three genetic clusters differed significantly from the random model (Tukey HSD: *p *< .0001 for all comparisons).

### 3.4 Interspecific variation in aggregative tendency

**Figure 6:**
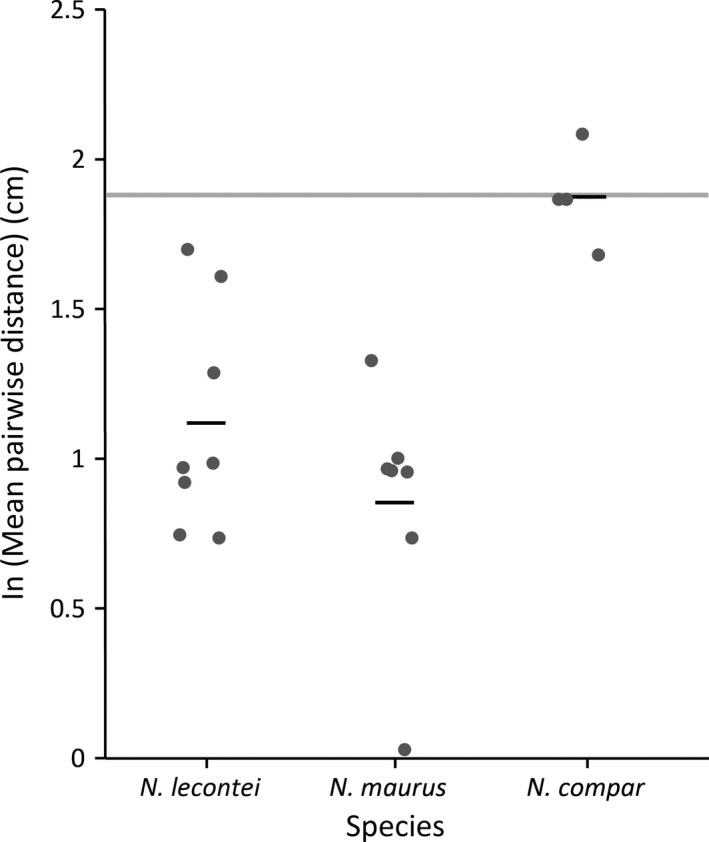
Interspecific variation in aggregative tendency. Each dark gray circle represents the log‐transformed (natural log) mean pairwise distances estimated from a single video of five late‐feeding instar larvae (following a 2,160‐s acclimation period); black bars represent the overall mean for each species (*Neodiprion lecontei*,* Neodiprion maurus*, and *Neodiprion compar*). The light gray bar represents the 95% confidence interval for the mean pairwise distance between five larvae estimated via simulation under a model of random larval distribution. Larvae of the two gregarious species, *N. lecontei* and *N. maurus*, were significantly more aggregative than the random model and larvae of the solitary species, *N. compar*

The three *Neodiprion* species we assayed differed significantly in their aggregative tendency (ANOVA, *F*_2,16_ = 10.6675; *p* = .0011). As expected, the two species that have previously been described as “gregarious” (*N. lecontei* and *N. maurus*) had significantly lower average pairwise distances than the “solitary” species, *N. compar* (Figure 6, Tukey HSD: *N. lecontei* vs. *N. compar*,* p* = .0085; *N. maurus* vs. *N. compar*,* p* = .0009). In contrast, the two gregarious species did not differ significantly from one another (Tukey HSD, *N. lecontei* vs. *N. maurus, p* = .3445). Consistent with these results, we found that the aggregative tendency of *N. compar* larvae was indistinguishable from the random model (Tukey HSD, *p* = .7534), while *N. lecontei* and *N. maurus* both exhibited a significant aggregative tendency (*N. lecontei* vs. random Tukey HSD: *p *< .0001; *N. maurus* vs. random Tukey HSD: *p *< .0001).

## 4 Discussion

Pine sawflies are a promising group of organisms for investigating both the proximate and ultimate causes of phenotypic variation. To facilitate future comparative and functional studies of one variable trait—larval gregariousness—we developed a quantitative assay of larval aggregative tendency. Using this assay, we tested several predictions regarding larval behavior in the highly gregarious pest species, *N. lecontei*. First, we found that while early‐ and late‐feeding instars do not differ appreciatively in their aggregative tendency, there is a pronounced shift in behavior in the last, nonfeeding instar. Second, we found that larval groups composed of kin do not have more cohesive aggregations than groups containing nonkin. Third, we found that variation in aggregative tendency among *N. lecontei* populations sampled across a wide geographical range is minimal compared to within‐population variation. Fourth, we found that our assay can be used to distinguish between species with “gregarious” larvae and those with “solitary” larvae. After discussing limitations of our assay, we discuss each of these findings and their implications for the causes and consequences of larval gregariousness.

### 4.1 Assay limitations

Although our assay provides an objective and repeatable way to discriminate between gregarious and solitary larval phenotypes, there was also a great deal of variation among groups sampled from a particular developmental stage and population (Figures 3 and 5). While this apparent “noise” may reflect true variation in aggregative tendency at the level of the individual or the group, it may also stem from developmental or sex‐based differences in behavior that we did not properly account for in our assays. First, in grouping the 5–6 feeding instars into two developmental stages (early‐feeding and late‐feeding), we may have lumped together behaviorally distinct instars. Second, behavioral differences in aggregative tendency between the sexes could have contributed to variation in our mixed‐sex assays (Figures 3 and 6). We note, however, that among‐group variation is also evident in single instar assays (final instars; Figure 3) and in male‐only assays (Figures 4 and 5). Third, behavioral variation may change over the course of a single instar. For example, larvae may exhibit differences in aggregative tendency as they prepare to molt or based on their physiological state such as hunger (Costa & Louque, 2001; Fletcher, 2015; McClure, Ralph, et al., 2011; Ribeiro, 1989; Tremmel & Muller, 2013). Future assays can account for some of these potential sources of variation via more precise developmental staging of larvae and, because larvae are difficult to sex, using male‐only colonies. Other potential sources of variation in our assays include fluctuations in light, temperature, and olfactory environment (e.g., stemming from presence of other larval colonies in the environmental room in which we recorded our videos) among videos. Regarding possible temporal effects, although we did not control for time of day in our assays, we note that previous work on *N. lecontei* larval activity patterns indicates that they lack a clear circadian pattern to their group foraging dynamics—instead, they feed continuously throughout the day and night (Flowers & Costa, 2003). Nevertheless, failure to account for potential sources of variation in larval aggregative tendency may have hampered our ability to detect small, but biologically meaningful, differences in larval behavior.

Another limitation of our assay is that larval aggregative behavior in our artificial assay environment (petri dish, no host, small groups) may not fully recapitulate behavior in the wild. First, our artificial, bounded arena may induce wall‐following behavior similar to that seen in cockroaches, *Blatella germanica* (Jeanson et al., 2003, 2005). Second, a lack of host material in the test arena also deviates from the natural conditions under which larvae aggregate. Our rationale for excluding host material was that we wanted to observe how larval interactions alone shape aggregative tendency. Because feeding larvae are attracted to host foliage, the presence of host material in a test arena might have caused otherwise solitary larvae to appear highly aggregative (Coppel & Benjamin, 1965; Ghent, 1960). Also, we note that host‐independent aggregations have been documented in nature—for example, *N. lecontei* larvae have been observed to migrate *en masse* up to 19 feet in search of a new host plant (Benjamin, 1955). Third, our experimental groups were much smaller than the *N. lecontei* groups that are typically encountered in the field (Benjamin, 1955; Costa & Louque, 2001; Wilson, 1977; (John W. Terbot II & Catherine R. Linnen, Personal observations)). If differences in gregariousness manifest as differences in preferred group size, we would not have detected these differences with our assay. Although additional work is needed to determine how different features of the environment or larval colony influence larval gregariousness, our results clearly indicate that even under our admittedly artificial assay conditions, we can reliably distinguish between aggregative and nonaggregative larvae (Figures 3 and 6).

Finally, because we assayed groups rather than individuals, we could not assess variation at the level of the individual. Our decision to assay behavior in groups was a practical one. Although we could isolate larvae and measure response to particular aggregation cues, we have not yet identified the pertinent cues. Moreover, isolated *N. lecontei* larvae become very agitated and exhibit increased wandering (Kalin & Knerer, 1977). Another alternative would have been to track individual larvae within a group. However, because behaviors of individual larvae in a test arena are not independent, the most appropriate unit of replication is the group (Costa & Louque, 2001).

### 4.2 Effects of developmental stage and relatedness on larval aggregative tendency

Despite the limitations of our assay, we found clear evidence that, following the final molt, larvae shift from a “gregarious” feeding mode to a “solitary,” nonfeeding mode. While these findings confirm previous natural history accounts, we introduce for the first time an objective criterion for categorizing larval behavior (i.e., via comparison to a random distribution). Similar behavioral shifts have been reported in many other insect taxa and are thought to facilitate dispersal to a suitable location for completing development (Dominick & Truman, 1984; Jones, Harwood, Bowen, & Griffiths, 1992; Li et al., 2016; Nijhout & Williams, 1974; Riemann, Beregovoy, & Ruud, 1986; Sedlacek, Weston, & Barney, 1996). There are two distinct mechanisms by which this developmental shift could occur: (1) final instar larvae may simply lose their attraction to conspecifics; or (2) final instar larvae may switch their response to conspecifics from attraction to repulsion. Intriguingly, our 5‐larvae assays indicated that final instar larvae maintain greater interlarvae distances than expected by chance, suggesting that they may be actively avoiding other larvae. Although the 8‐larvae final instar data trended in the same direction, the departure from random expectations was not significant. One explanation for this apparent discrepancy is that, within a test arena of fixed size, larger groups of larvae cannot spread out enough to distinguish between a random distribution and an overdispersed one.

Our data also indicate that, in contrast to late‐feeding instars of some *Neodiprion* species (Anstey et al., 2002; Furniss & Dowden, 1941; Hetrick, 1956; Hopping & Leech, 1936; Rose & Lindquist, 1994), late‐feeding instars of *N. lecontei* do not exhibit a pronounced reduction or loss of gregariousness. These results imply that larval aggregations remain beneficial throughout the larval feeding period of *N. lecontei*. Costs and benefits of larval aggregations are also relevant to whether or not cooperative behaviors should be directed preferentially to kin. If behaviors are costly to the individuals, kin‐based behavioral preferences are more likely to evolve (Hamilton, 1964). In contrast to these predictions, we did not observe any detectable reduction or loss of gregariousness in larval groups that contained nonkin. Similarly, fusion of unrelated *Neodiprion* colonies—and even different *Neodiprion* species—appears to be relatively common in nature, especially at high population densities (Codella & Raffa, 1993, 1995a; Costa & Louque, 2001; Tostowaryk, 1972; personal observation). These observations suggest that, when it comes to forming and maintaining larval aggregations, larvae do not discriminate between kin and nonkin (Figure 4). These findings cannot be explained by a lack of capacity for kin recognition in this species because previous work has shown that adults do discriminate between related and unrelated mates (Harper et al., 2016). One possible implication of our results is that individuals derive sufficient direct benefits from aggregating that kin selection need not be invoked to explain the evolution and maintenance of larval gregariousness.

That said, there could be opportunities for larvae to discriminate against nonkin in ways that we would not have detected in our assays. For example, in small colonies, *N. lecontei* larvae continually cycle between exposed and protected feeding positions (Codella & Raffa, 1993). Also, colony defense is enhanced by simultaneous regurgitation of host resin—both by startling would‐be predators (Sillén‐Tullberg, 1990) and coating one another with sticky regurgitant that increases handling time (Eisner, Johnessee, Carrel, Hendry, & Meinwald, 1974; Tostowaryk, 1972). Because assuming exposed positions and depletion of larval defenses can be costly (Higginson, Delf, Ruxton, & Speed, 2011; Miettinen, 2015), unrelated individuals may be less willing to incur these costs. Thus, analysis of the effect of relatedness on position cycling and defensive regurgitation may reveal these subtler forms of kin discrimination in feeding *N. lecontei* larvae.

### 4.3 Intra‐ and interspecific variation in larval aggregative tendency

One approach that has been used to investigate adaptive function of particular traits is to see how phenotypic variation within species correlates with environmental variables (Garland & Adolph, 1994; Harvey & Pagel, 1991; Pulliam & Caraco, 1984; Reeve & Sherman, 1993). For example, latitudinal variation in color and diapause characteristics—both of which are important adaptations to different temperature regimes—are widespread in nature (Alho et al., 2010; Chahal & Dev, 2013; Lehmann, Lyytinen, Piiroinen, & Lindström, 2015; Masaki, 1999; Parsons & Joern, 2014). Likewise, there is empirical evidence from several organisms that feeding aggregations can improve thermoregulation (Codella & Raffa, 1993; Fletcher, 2009; Joos et al., 1988; Klok & Chown, 1999; Seymour, 1974). If aggregations serve a thermoregulatory function in *N. lecontei*, there may be clinal variation in aggregative tendency. Although we have surveyed only a small number of *N. lecontei* populations, the populations we did sample were distributed across a broad latitudinal gradient—if there was clinal variation, we would expect to see differences among the latitudinal extremes (e.g., FL vs. MI). In contrast to these predictions, we found that larvae from all populations aggregated significantly more than expected under the random model and that variation among groups within populations exceeded variation among populations (Figure 5).

Nevertheless, with these data alone, we cannot rule out a thermoregulatory function for larval aggregations. For example, it is possible that these populations differ in their plastic responses to temperature (e.g., perhaps northern populations show increased aggregation at low temperatures)—such a difference would not have been detected in our assays, which took place at a single temperature. Thus, to definitively assess latitudinal variation in aggregative behavior, these assays should be repeated at different temperatures.

In contrast to the lack of variation among populations, we observed pronounced differences among species: Whereas *N. lecontei* and *N. maurus* are decidedly gregarious, *N. compar* larvae are not. The pronounced difference between *N. lecontei* and *N. compar* is especially intriguing because these two species have a very similar geographical distribution, share many of the same hosts, and contend with many of the same predators and parasites (Linnen & Farrell, 2010). The most obvious difference between these two species is in their larval coloration: Whereas *N. lecontei* larvae are conspicuously colored (white to bright yellow body with several rows of spots), *N. compar* larvae are cryptically colored (green body covered by longitudinal green stripes). One possible explanation for this association between gregariousness and conspicuous coloration is that larval aggregations amplify aposematic signals, thereby enhancing avoidance learning and reducing predation (Riipi, Alatalo, Lindström, & Mappes, 2001). Consistent with this hypothesis, phylogenetic analyses of folivorous lepidopterans that suggest that aposematic coloration often evolves before gregariousness (Beltrán, Jiggins, Brower, Bermingham, & Mallet, 2007; Sillen‐Tullberg, 1988; Tullberg & Hunter, 1996). While our results suggest that a similar trend may also be true for pine sawflies, evaluating this hypothesis will require comparable data from more *Neodiprion* species and a formal phylogenetic comparative analysis.

## 5 Conclusions

Although much work remains, our results have several implications for the proximate and ultimate mechanisms underlying larval aggregations in *Neodiprion*. From a proximate perspective, we have shown that larval grouping occurs even in the absence of host plant material, highlighting the importance of cues from the larvae themselves. Nevertheless, in terms of the maintenance of these aggregations, there does not appear to be any sort of kin discrimination in the feeding larvae. We also describe how gregarious behavior changes over the course of *N. lecontei* larval development. From an ultimate perspective, our observation that larvae remain gregarious throughout the feeding period and that larvae do not discriminate against nonkin suggests that, in *N. lecontei*, the benefits of aggregating to the individual consistently outweigh the costs. Moreover, as we have demonstrated here, this assay can be applied to any *Neodiprion* species. Future work will examine the costs and benefits of aggregations over different developmental stages and multiple species using both experimental and comparative approaches. Together, these data will provide a comprehensive understanding of aggregative behavior.

## Conflict of Interest

None declared.
